# Comparing reliability-based measures of functional connectivity between movie and rest: An ROI-based approach

**DOI:** 10.1162/imag_a_00411

**Published:** 2025-01-02

**Authors:** Hallee Shearer, Jeffrey Eilbott, Fidel Vila-Rodriguez, Stephanie Noble, Ting Xu, Tamara Vanderwal

**Affiliations:** Department of Psychiatry, University of British Columbia, Vancouver, BC, Canada; School of Biomedical Engineering, University of British Columbia, Vancouver, BC, Canada; Department of Psychology, Northeastern University, Boston, MA, United States; Department of Bioengineering, Northeastern University, Boston, MA, United States; Center for Cognitive and Brain Health, Northeastern University, Boston, MA, United States; Department of Brain Development, Child Mind Institute, New York, NY, United States; BC Children’s Hospital Research Institute, Vancouver, BC, Canada

**Keywords:** precision psychiatry, naturalistic neuroimaging, functional connectivity, region of interest, test–retest reliability, discriminability

## Abstract

Functional connectivity (FC) has shown promising utility in the field of precision psychiatry. However, to translate from research to clinical use, FC reliability and sensitivity to individual differences still require improvement. Movie watching as an acquisition state offers advantages at the whole-brain level that align with the requirements of FC for individualized measures. However, it is unclear whether these advantages hold in specific brain regions important for precision psychiatry. Here, we compared univariate and multivariate reliability-based measures of movie-watching and resting-state FC data in three psychiatrically relevant brain regions. We found that the reliability of movie-watching FC was comparable with resting-state FC in the dorsolateral prefrontal cortex and presupplementary motor area, and movie-watching FC was more discriminable than resting-state FC in the temporoparietal junction. Rest had higher reliabilities at lower data amounts (e.g., under 5 minutes of scan time). We then expanded this approach to all brain regions and showed that for image intraclass correlation coefficients (I2C2), no parcels were significantly different between movie and rest. For discriminability, 25% (94/379) of parcels were better for movie than for rest, and zero parcels were better for rest. For fingerprinting, 59 parcels were better for movie (mainly in visual and temporal regions, mean improvement in accuracy = 23%) and 4 parcels were better for rest. For researchers interested in cross-state differences in FC reliability, we provide an interactive visualization tool that displays the results for all measures and for all regions in both movie and rest. These findings suggest that movie watching as an acquisition state—even when using different movies across scans—may provide a useful alternative to resting state in research studies that require optimization of FC discriminability.

## 1 Introduction

Precision psychiatry aims to use biologically based data from an individual patient to improve diagnosis and treatment of psychiatric diseases (Fernandes et al., 2017;Gómez-Carrillo et al., 2023;Gratton et al., 2020;Laumann et al., 2023). This includes both foundational biomarker approaches that could lead to useful clinical tests and other levels such as genetics. At the crux of all these research efforts in functional Magnetic Resonance Imaging (fMRI) is a shift from studying patients at the group level to the individual level, which almost always requires large amounts of high-quality data (i.e., longer and/or more frequent scans). Particularly with functional connectivity (FC)-based fMRI studies, significant challenges still exist regarding data quality and reliability, especially when long scans are required.

### 1.1 FC-based precision psychiatry

In psychiatry research, ongoing efforts are attempting to use individual-level FC to identify personalized targets for neurostimulation interventions (Cash, Cocchi, et al., 2021;Cash, Weigand, et al., 2021;Elbau et al., 2023;Fox et al., 2013;Ji et al., 2021;Siddiqi et al., 2020,^2023^), to identify disorder subtypes (Drysdale et al., 2017;Xia et al., 2018), and to investigate neural correlates of risk for psychiatric illness (Clark et al., 2018;Harnett et al., 2021). Precision mapping has expanded on these efforts in a unique way. By collecting large amounts of fMRI data in small number of subjects (i.e., dense sampling), researchers have demonstrated that high-fidelity individual-specific connectomes can be resolved (DiNicola et al., 2023;Gordon et al., 2017;Laumann et al., 2015;Poldrack, 2017;Poldrack et al., 2015;Sylvester et al., 2020). These individual-level FC maps are reliable and reveal information that is obfuscated by group averaging (Gordon et al., 2017;Poldrack et al., 2015;Sylvester et al., 2020).

Despite these advances and pressing clinical needs, these endeavors have yet to translate into clinical use.Gratton et al. (2020)posit that clinical translation of FC-based findings is hindered by intersubject heterogeneity (both clinical and neural) and low reliability of FC measures. They suggest that new methods are needed to improve the reliability, sensitivity to individual differences, quality, and quantity of FC data to improve our chances at achieving clinical translation.

While there is currently a necessary focus on improving study design and analytic approaches in FC-based research to address these limitations, such as multiecho fMRI (Lynch et al., 2021), optimizing echo time parameters (Vasileiadi et al., 2023), and dense sampling of individual subjects (Poldrack, 2017), relatively little attention has been paid to the acquisition state being used, and most studies have relied exclusively on the use of task-free resting-state conditions. Acquisition state may offer a unique opportunity to systematically constrain some forms of variability, to reduce noise, and, therefore, to facilitate the detection of a useful, reliable signal.

### 1.2 Movie-fMRI as an acquisition state for FC research

**Fig. 1.**
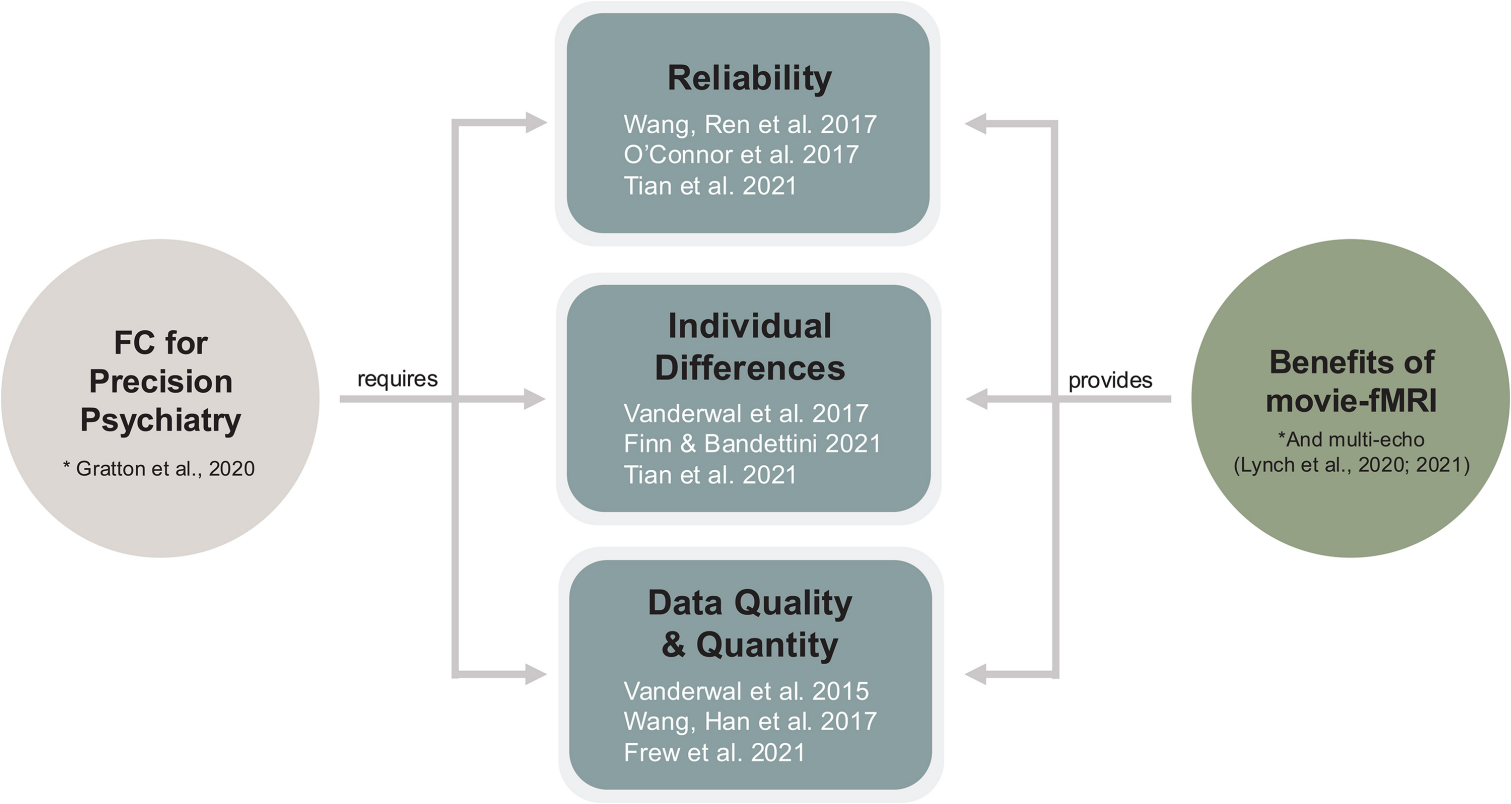
Overlap between the requirements of FC for precision psychiatry and the benefits of movie-fMRI.

Movie-fMRI features the use of movies as stimuli during functional runs. This approach within functional connectivity research has been motivated by the desire to investigate more ecologically valid neural patterns (Nastase et al., 2020;Sonkusare et al., 2019;Vanderwal et al., 2019) and has since been propelled by findings of multiple advantages of movie watching when compared with resting-state and traditional task scans. The current paper was based on the observation that many of these advantages align with the challenges facing FC-based precision psychiatric research (Fig. 1).

#### 1.2.1 Advantages of movie-fMRI for FC test–retest reliability

Test–retest reliability analyses estimate the within-subject stability of a measure across repeated measurements. Reliability is a fundamental requirement for potential biomarkers, and for any brain measure to be clinically useful. Specifically in studies of FC during movie-fMRI, Wang et al. used scan-wise Intra-Class Correlations (ICCs), averaging FC across all edges before calculating the ICC, to show that FC has higher whole-brain reliability across identical movie scans (ICC = 0.7593) than rest scans (ICC = 0.5381) (Wang, Ren, et al., 2017). Other studies have reported similar ICCs from movie FC to rest FC (O’Connor et al., 2017;Tian et al., 2021), possibly due to novelty effects across repeated movie viewings (O’Connor et al., 2017), or the use of different movies across scans (Tian et al., 2021). Further work is needed to better understand FC reliability with specific movies, in specific brain regions, and in patient populations.

#### 1.2.2 Advantages of movie-fMRI for the reliable detection of individual differences in FC

Connectome fingerprinting can be utilized to consider the reliability of individual differences in FC (Finn et al., 2015). Essentially, this algorithm operationalizes the ratio of between-subject variance and within-subject similarity at the whole-brain level. Across resting-state scans, fingerprinting has achieved accuracies as high as 94.4% (Finn et al., 2015). Applying the fingerprinting algorithm to movies,Vanderwal et al. (2017)found 100% accuracy using movies (albeit in a smaller sample) and showed that cross-movie identification also had high accuracies even when two markedly different movies were used and scan sessions were 1-week apart. This high cross-movie finding was recently replicated in a larger HCP sample (N = 178) in which individuals were identified based on their FC matrices across four different movie scans with accuracies of 94–99% (Tian et al., 2021).

#### 1.2.3 Advantages of movie-fMRI for data quality and quantity

Data quality and quantity are threatened by head motion and arousal levels. Movie-fMRI dramatically reduced head motion in some age ranges, enabling improved data quality and quantity in these difficult-to-scan populations (Frew et al., 2022;Greene et al., 2018;Vanderwal et al., 2015). Specifically, movies appeared to prevent the linear increase in head motion that usually occurs within a run, suggesting that movie-fMRI may be of particular help in obtaining longer scans such as those needed for dense sampling efforts (Frew et al., 2022). The use of movies also decreased self-reports of sleeping during functional runs relative to rest in healthy adults (Vanderwal et al., 2017), and when tracked with heart-rate variability, movies mitigated the effects of drowsiness-induced changes in FC (Wang, Han, et al., 2017).

### 1.3 Movie-fMRI for FC-based precision psychiatry

The majority of movie-fMRI studies that have quantified reliability and individual differences have reported results across the whole brain or within networks, yet these measures are known to vary regionally throughout the brain (Finn et al., 2015;Noble et al., 2017;Shah et al., 2016). Precision psychiatric research is often focused on small regions or even smaller specific FC-based targets (especially for research in interventional psychiatry), but it is unknown whether the advantages of movie-fMRI shown at the whole-brain and network level will apply to small regions of the brain relevant to precision psychiatric research. While some studies graphically display edge-level and network-level results (Gao et al., 2020;Tian et al., 2021;Vanderwal et al., 2017;Wang, Ren, et al., 2017), they do not report results by region and only report average univariate results or whole-brain results. The present study aims to fill this knowledge gap by comprehensively comparing movie and resting-state FC data in three brain regions of interest (ROIs) for FC-based precision psychiatry: the left Dorsolateral Prefrontal Cortex (DLPFC), the left Temporoparietal Junction (TPJ), and the right pre-Supplementary Motor Area (pre-SMA), using a full array of univariate and multivariate reliability-based metrics and maintaining fine-grained or vertex-level values as much as is possible.

These three ROIs were selected a priori due to their relevance in psychiatric research. The DLPFC has been repeatedly implicated in multiple psychiatric disorders, and especially in Major Depressive Disorder (Balderston et al., 2017;Koenigs & Grafman, 2009;Li et al., 2020;Potkin et al., 2009). The TPJ is of particular interest in psychotic disorders (Clos et al., 2014;Eddy, 2016;Penner et al., 2018;Vercammen et al., 2010) and the pre-SMA has been linked to Obsessive-Compulsive Disorder (OCD) and tic-related disorders, among others (De Wit et al., 2012;Ganos et al., 2014;Yücel et al., 2007). More specifically, these ROIs were selected to reflect a practical application of FC-based precision psychiatry in which reliability is a pressing concern: individualized FC-guided repetitive Transcranial Magnetic Stimulation (rTMS) targeting. Previous research has explored the potential of individualized FC targets for rTMS treatments in each of these three ROIs: the left DLPFC for depression, the left TPJ for auditory verbal hallucinations, and the right pre-SMA for OCD (Briend et al., 2020;Cash, Cocchi, et al., 2021;Fox et al., 2013;Ji et al., 2021;Sommer et al., 2018).

Here, we used publicly available resting-state (Rest) and movie-watching (Movie) data in healthy adults from the HCP dataset to investigate differences in univariate and multivariate reliability of FC between Movie and Rest in three psychiatrically relevant ROIs. The goal was to explore whether the advantages of movie-fMRI, previously demonstrated at the whole-brain and network level, also apply to brain regions of interest for precision psychiatry. We then applied this high-resolution ROI-level test–retest reliability approach to all brain parcels, including subcortical regions, to inform decisions surrounding acquisition state for a broader array of future FC studies.

## 2 Methods

### 2.1 Dataset

All data are from the HCP 7T release (Van Essen et al., 2013). Data from a total of 184 healthy adult participants were collected during rest and while watching movie clips at the University of Minnesota with a Siemens Magnetom scanner (TR = 1000 ms, seehttps://www.humanconnectome.org/hcp-protocols-ya-7t-imagingfor parameters). Resting state was eyes open with a bright fixation cross on a dark background. Movie stimuli contained three shorter movie clips (3:48 to 4:19 minutes long) separated by 20 seconds of rest and a 1:23 minute validation clip (common across movie runs) at the end. We will refer to the condition of watching concatenated movie clips in the scanner as Movie and resting state as Rest. Four Movie runs and four Rest runs were available. We selected only the two Movie runs that contained Hollywood movie clips (as opposed to Creative Commons) as they are more engaging and cinematically quite different from the Creative Commons clips. We then selected the two Rest runs with phase encoding that matched the Hollywood movie runs. Therefore, we used Rest1, Rest4, Movie2, and Movie4 (each 15–16 minutes long). Rest1 and Movie2 (with posterior–anterior phase encoding) were collected on the first day, and Rest4 and Movie4 (with anterior–posterior phase encoding) on the second day. Written informed consent was obtained from each subject by the HCP committee and approved by the Washington University Institutional Review Board (Van Essen et al., 2013).

### 2.2 Participants

Participants were healthy young adults between the ages of 22 and 36 years old. This dataset included sets of mono- and dizygotic twins, as well as siblings. After excluding 73 subjects with mean framewise displacement greater than 0.2 mm in any functional run, and 2 subjects based on quality control checks (unusually low correlation between Rest1 and Rest4 FC matrices), our sample included 109 subjects with a mean age of 29.4 ± 3.4 years and 64 females (24 sets of monozygotic twins, 14 sets of dizygotic twins, 4 siblings, 29 unrelated).

### 2.3 FC Matrices

HCP minimally preprocessed data were used (Glasser et al., 2013), for which no slice time correction was performed, spatial processing was applied, motion was corrected with FLIRT-based motion correction (no motion censoring was performed, subjects with mean FD > 0.2 mm were excluded), and structured artifacts were removed using ICA + FIX (independent component analysis followed by FMRIB’s ICA-based X-noisifier). Data are represented as a time series of grayordinates in CIFTI format (i.e., cortical surface vertices and subcortical standard-space voxels). The first 30 TRs of each scan were removed, as well as 20-second epochs, and 10 TRs after each epoch, of rest that occurred between clips in the movie scans. We also removed temporally corresponding epochs from rest scans (20 seconds and the 10 TRs after each epoch). The runs were cropped to match the length of runs within each day. The resulting Rest1 and Movie2 scans were 704 TRs in length, and Rest4 and Movie4 scans were 687 TRs in length. The mean framewise displacement (FD) for these runs was 0.11 mm for Rest1, 0.11 mm for Movie2, 0.14 mm for Rest4, and 0.12 mm for Movie4. Importantly for our test–retest purposes, the differences in FD from Rest1 to Rest4 (0.023) and from Movie2 to Movie4 (0.017) were not significantly different (p = 0.12).

Using Connectome Workbench (Marcus et al., 2013), we created ROI masks based on the Glasser parcellation for the left DLPFC, left TPJ, and right pre-SMA. For each ROI, we used the suggested parcels for each mask as described byGlasser et al. (2016).

The time courses of all vertices within these masks were extracted, and the cortex was parcellated into 360 parcels (Glasser et al., 2016). The 19 subcortical regions extracted with the HCP minimal preprocessing pipeline were included (Glasser et al., 2013), producing a total of 379 parcels. For each ROI, FC matrices of ROI vertices by parcels (DLPFC matrix: 2207 vertices x 379 parcels, TPJ matrix: 722 vertices x 379 parcels, pre-SMA matrix: 209 vertices x 379 parcels) were computed using Pearson correlations between paired time courses for each subject (109 subjects) and condition (Movie2, Movie4, Rest1, Rest4).

This approach was also repeated for all parcels of the brain, treating each Glasser parcel and subcortical region as an ROI. For this analysis, the resulting FC matrices contained the Pearson correlations between the parcel’s vertices (or voxels for subcortical regions) and the 379 parcels (360 Glasser + 19 subcortical).

### 2.4 Test–retest reliability

Test–retest reliability is the consistency of a measure across repeat samplings. The most common measure of univariate reliability in fMRI is the ICC. ICCs can be averaged across the brain or across a region of interest (ROI); however, multivariate measures have been introduced to better account for the high dimensionality of fMRI data, such as the Image Intraclass Correlation Coefficient (I2C2), discriminability (Discr), and fingerprinting. Of note, there is no “pure” or definitive measure of reliability, and each of these measures captures different aspects that relate to test–retest reliability. The concepts of test–retest reliability and identifiability are closely linked, and discriminability and fingerprinting provide insight into the identifiability of the data. Of note, a highly reliable measure may have very little subject identifiability, but identification requires reliability. Test–retest reliability also does not stand alone in a practical sense, and factors that affect data quality, such as head motion and acquisition and scanning parameters, can influence test–retest reliability in an intricate way (Noble et al., 2017,^2019^). Multivariate reliability of FC has been shown to be higher than univariate reliability (Camp et al., 2024;Noble et al., 2017), but multivariate measures do not suggest homogeneity of the reliability at the univariate level. In other words, when multivariate reliability of a brain region is high, there may still be parts of the region that have low reliability (Xu et al., 2022).

#### 2.4.1 Image intraclass correlation coefficient

I2C2 is a nonparametric, multivariate generalization of the ICC (Shou et al., 2013). The I2C2 is a more appropriate multivariate measure of reliability for an image than the mean ICC across variables. The I2C2 estimates the proportion of the total variability that arises from the subject level, specifically by using a multivariate image measurement error model. We used the R package ReX (Xu et al., 2023) to calculate I2C2 values of the FC for each condition from the Euclidian distance matrix of the distances within and between scans. The I2C2 value is estimated by

$$I2C2=1-\frac{trace\left(K_{u}\right)}{trace\left(K_{o}\right)},$$

where

$$trace\left(K_{o}\right)=\frac{1}{\sum_{i}J-1}\sum_{i}\sum_{j}\sum_{v}{\left(X_{ij}\left(v\right)-X..\left(v\right)\right)}^{2}$$

and

$$trace\left(K_{u}\right)=\frac{1}{\sum_{i}\left(J-1\right)}\sum_{i}\sum_{j}\sum_{v}{\left(X_{ij}\left(v\right)-X._{i}\right)}^{2}.$$

In these equations,X..(v)is the average over all individuals and all repetitionsJfor each variablev , and$X._{i}$is the average over all repetitionsjfor each individualiand variablev .

#### 2.4.2 Discriminability

Discriminability represents how relatively similar an individual’s repeated measurements are to each other and consequently can provide a lower bound on subsequent classification accuracy (Bridgeford et al., 2021). Data that are highly discriminable contain measurements that are more similar within subjects than across subjects. Of note, the discriminability statistic is multivariate, like the I2C2, but does not assume the data to be Gaussian or Euclidean. The discriminability statistic compares the rank of the Euclidian distances between a subject’s repeated scans with the distances between one subject and another subject’s scans. The discriminability of FC was computed for each condition with ReX (Xu et al., 2023), as the fraction of times that the observed within-individual distance (e.g., subject 1, scan 1 to subject 1, scan 2) was greater than the between-individual distances (e.g., subject 1, scan 1 to every other subject’s scan 2) (Bridgeford et al., 2021).

#### 2.4.3 Fingerprinting

Connectome fingerprinting is a multivariate test–retest reliability metric similar to discriminability; however, fingerprinting accuracy represents binary successful or unsuccessful identifications across participants (Finn et al., 2015;Vanderwal et al., 2017). A successful match represents both reliability and intricacies of FC that make an individual’s matrix distinct from matrices of other subjects. Fingerprinting accuracy was computed with ReX (Xu et al., 2023) for each condition as the fraction of subjects for which observed within-individual distance was smaller than all between-individual distances. In other words, the fingerprinting accuracy is the fraction of subjects who can successfully be identified across scans by the similarity of their scans.

Discriminability and fingerprinting are similar measures, as they both consider the intra- and interindividual similarity in measurements and are both also related to identifiability (Xu et al., 2022). However, fingerprinting is more binary than discriminability: if a subject’s two matrices are not most similar to each other but instead are more similar to a different subject’s matrix, this counts as a binary negative against the identification accuracy. This negative is equal in magnitude whether the subject’s two matrices were the second most similar or whether they were the least similar. On the contrary, discriminability utilizes more of a continuum by representing the fraction of times that across-individual measurements are closer in similarity (smaller distance) than within-individual measurements.

#### 2.4.4 Intraclass correlation

ICC is a parametric, univariate measure of test–retest reliability that represents the ratio of between-individual variance and total variance. We calculated the ICC(2,1), also referred to as ICC(A-1), of each edge of the functional connectivity matrices (between ROI vertices and brain parcels) for each ROI and condition with MATLAB’s ICC function (Salarian, 2024). This function calculates the ICC as

$$ICC=\frac{\left(MSR-MSE\right)}{(MSR+\left(k-1\right)*MSE+k*\frac{\left(MSC-MSE\right)}{n}},$$

where MSR is the mean square row (between-subject), MSE is the mean square error, MSC is the mean square column (between-measurements), k is the number of observations, and n is the number of subjects. SeeMcGraw & Wong (1996)for a thorough explanation of the ICC model.

We averaged the ICC values across brain parcels for each vertex to find the average ICC value for each vertex in the ROI. For example, we calculated the ICC values for each edge in the FC matrix of pre-SMA vertices to all Glasser parcels of the brain (Glasser et al., 2016). For each vertex in the pre-SMA, we then had multiple ICC values: one for each connection to each parcel of the brain. Therefore, we averaged across the parcels of the brain to obtain one ICC value for each vertex of the pre-SMA.

To provide a coarse estimate of ICC at the group level, and to enable comparisons from the univariate to multivariate measures, we averaged across vertices in each ROI to determine the average ICC for each condition and ROI, which were then compared across conditions.

#### 2.4.5 Statistical comparison

Test–retest reliability measures were compared between Movie and Rest for each ROI with nonparametric permutation testing. For multivariate measures (I2C2, Discriminability, Fingerprinting), condition labels were permuted at the level of the distance matrices. Permuted condition labels were created by randomly switching the rest and movie labels of each subject’s scans with a 50% probability. To be conservative, the nested structure of the data was maintained: the two scans of each condition always remained grouped, even if their collective labels switched from one condition to the other, so that the permutations were never measuring reliability across conditions (e.g., Movie to Rest). The permuted condition labels were used to recalculate each multivariate reliability measure for Movie and for Rest, and then the difference between conditions (Movie–Rest) was calculated. This process (shuffle labels, recalculate reliability, calculate difference) was repeated 5000 times for multivariate measures, and 500 times for ICC (due to computational limitations). The distribution of the permutations for a given measure represents the null distribution (i.e., the distribution of the expected differences between Movie and Rest if there is no true difference between conditions). The p-value for each measure was computed as the proportion of all permutations with an absolute value of the difference between conditions larger than the absolute value of the observed difference between conditions.

Differences in mean ICC were statistically compared between conditions in the same way, except that condition labels were permuted at the level of the FC matrices rather than distance matrices (as the ICC is not based on distances).

All p-values were FDR corrected to account for multiple comparisons. For the 3 psychiatrically relevant ROIs, p-values were corrected for 12 tests (3 ROIs, 4 measures). For the whole-brain analysis, p-values were corrected for 1,137 tests (379 parcels, 3 multivariate measures). Corrected p-values less than 0.05 were reported as statistically significant.

#### 2.4.6 Effect of scan duration

The three psychiatrically relevant ROI FC matrices were recreated with varying amounts of data. Starting with the first 20 TRs, we created matrices with 20 TR increments, the largest being 680 TRs. With these matrices, we reran all analyses at each amount of data.

## 3 Results

### 3.1 Region-specific advantages of movie-fMRI

**Fig. 2.**
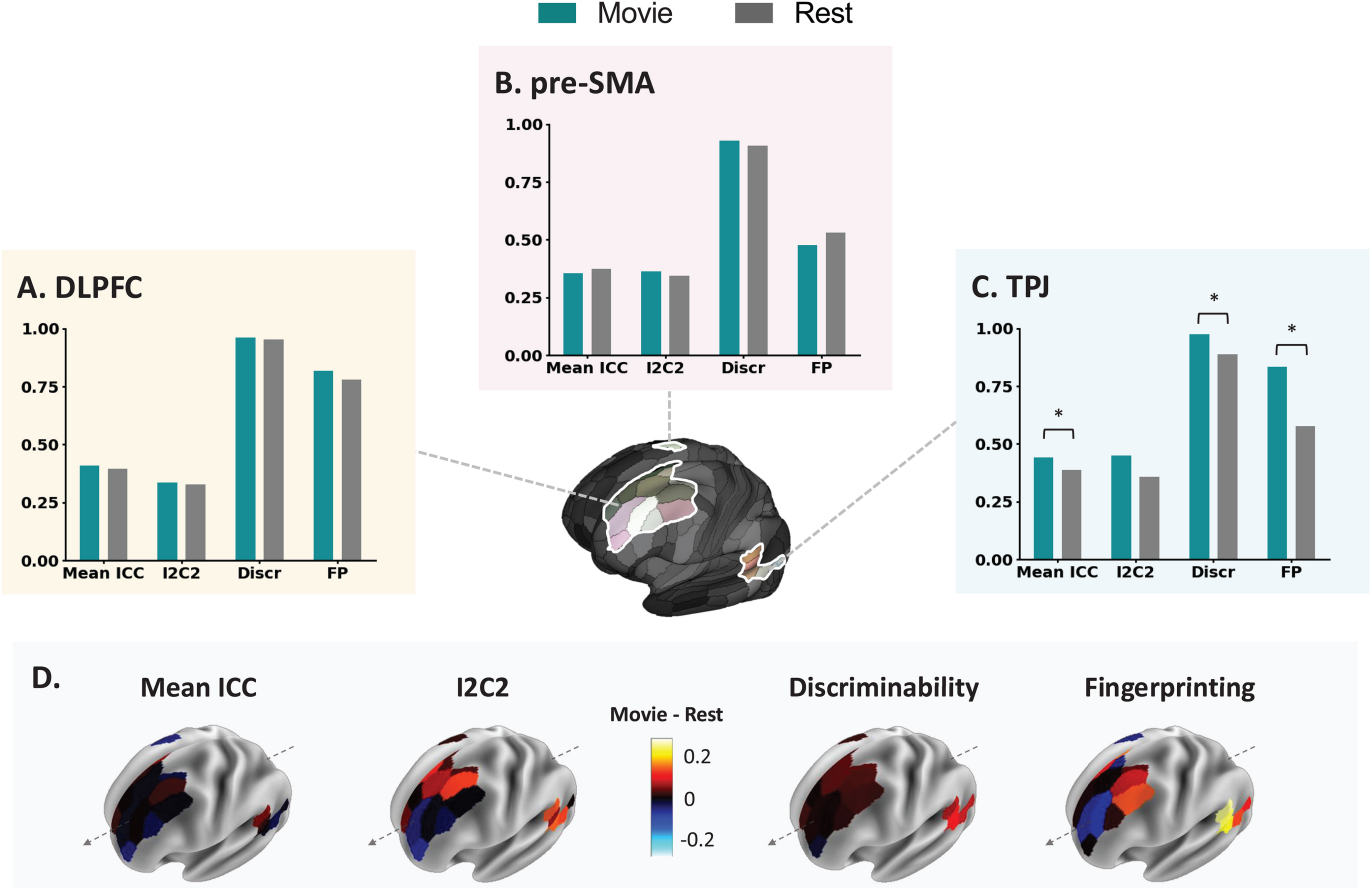
ROI-based reliability in Movie and Rest. All measures were comparable between Rest and Movie for the DLPFC (A) and pre-SMA (B). Mean ICC, discriminability, and fingerprinting were improved with movies in the TPJ (C). Star denotes significance of p < 0.05 (FDR corrected across all 12 tests), obtained from permutation testing. These results suggest region-specific advantages for Movie and no advantages in these regions for Rest. The spatial distributions of these measures are also visualized at the parcel level within each ROI (D). The y-axis of each graph represents different units for each measure: mean ICC value, I2C2 value, discriminability value, and fingerprinting accuracy (from left to right). Discr = discriminability, FP = fingerprinting.

Four estimates of test–retest reliability were calculated for each ROI and condition (Fig. 2). ICC values are commonly grouped into the following categories: poor (ICC < 0.2), fair (0.2 < ICC < 0.39), moderate (0.4 < ICC < 0.59), good (0.6 < ICC < 0.79), and excellent (0.8 < ICC) (Cicchetti & Sparrow, 1981). Mean ICC values were fair across all conditions and ROIs, except the TPJ and DLPFC during Movie, which reached moderate ICC values of 0.453 and 0.414, respectively. There were no meaningful differences in mean ICC values between Movie and Rest in the pre-SMA (Movie = 0.0.365, Rest = 0.0.374, adjusted p = 0.82) and DLPFC (Movie = 0.414, Rest = 0.398, adjusted p = 0.73). For the TPJ, mean ICC values were significantly higher with Movie (0.453) than with Rest (0.391; adjusted p = 0.016).

I2C2 estimates were fair, except for TPJ Movie (0.45), which was moderate. I2C2 was comparable between Movie and Rest for all regions (DLPFC: Movie = 0.34, Rest = 0.33, adjusted p = 0.96; TPJ: Movie = 0.45, Rest = 0.36, adjusted p = 0.73; pre-SMA: Movie = 0.36, Rest = 0.34, adjusted p = 0.94). All discriminability estimates were high, ranging from 0.89 for TPJ Rest to 0.98 for TPJ Movie. Discriminability was comparable between Movie and Rest for the DLPFC (Movie = 0.96, Rest = 0.95, adjusted p = 0.77) and pre-SMA (Movie = 0.93, Rest = 0.91, adjusted p = 0.71), and higher in Movie for the TPJ (Movie = 0.98, Rest = 0.89, adjusted p = 0.00). Fingerprinting accuracies were highest in the DLPFC and lowest in the pre-SMA. Fingerprinting was comparable between Movie and Rest for the DLPFC (Movie = 0.82, Rest = 0.78, adjusted p = 0.77) and pre-SMA (Movie = 0.47, Rest = 0.53, adjusted p = 0.73), and higher for Movie than for Rest for the TPJ with a large effect size (Movie = 0.84, Rest = 0.58, adjusted p = 0.00). We also note that spatial heterogeneity across the parcels of the DLPFC was observed with I2C2 and fingerprinting, where more posterior parcels had more improvement with Movies than more anterior parcels (Fig. 2D).

### 3.2 Interaction between scan duration and condition

**Fig. 3.**
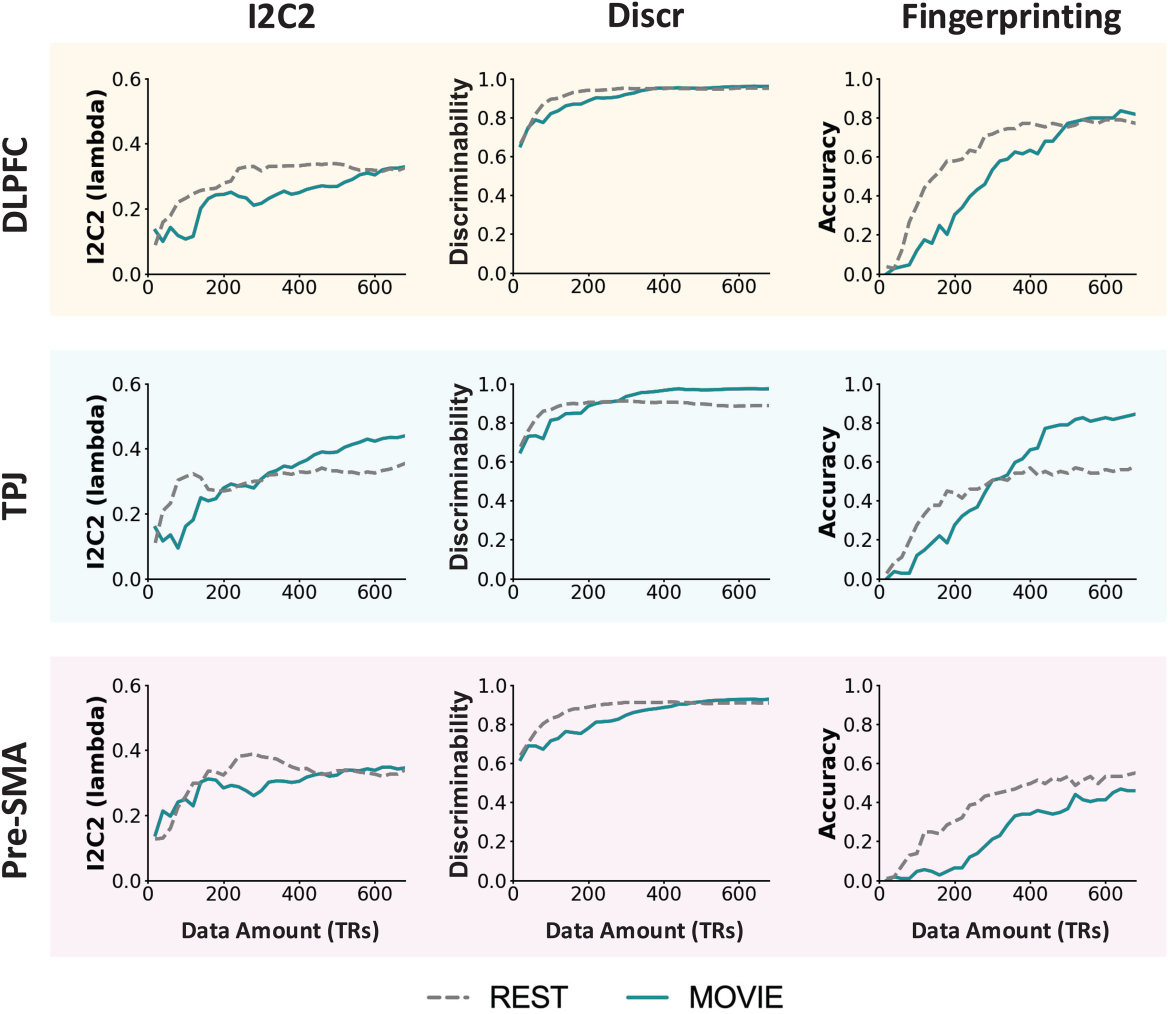
The effect of data amount on univariate and multivariate reliability. All measures improved with increasing data amount across Rest and Movie. Rest and Movie improved at similar rates; however, Rest improved faster at lower data amounts, and in the TPJ, Movie continued to improve after Rest reached a plateau.

We repeated the previous analyses with increasing scan durations from 20 TRs (TR = 1 second) to 680 TRs, with 20 TR increments (Fig. 3). All measures improved with increasing amounts of data across Rest and Movie; however, the pre-SMA improved more slowly than the DLPFC and TPJ. I2C2 was low for both conditions and improved slowly with increasing data amount. Discriminability was high even with a small number of TRs, and rapidly reached a plateau for both Movie and Rest. Fingerprinting improved quickly with increasing data in the DLPFC and TPJ. Across all measures, the reliability of FC from the TPJ continued to improve with Movie data after Rest reached a plateau. At low data amounts (i.e., less than 5–6 minutes), Rest measures seem to improve more rapidly than Movie.

### 3.3 Spatial distribution of ICCs within ROIs between Movie and Rest

**Fig. 4.**
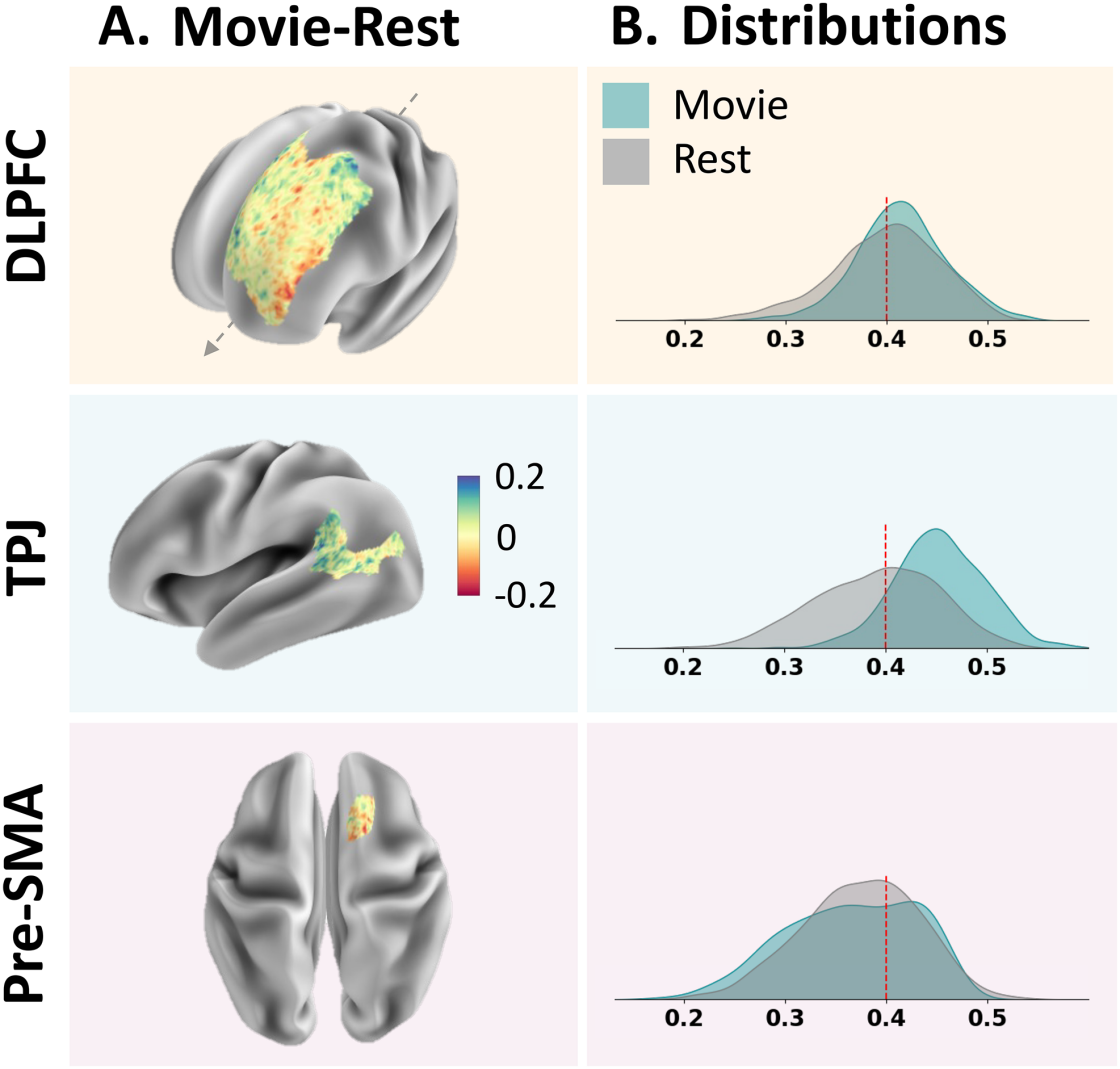
Vertex-level ICC values for each ROI. (A) Differences in ICC values between Movie and Rest were spatially diffuse within each ROI. Higher ICC with Movie shown in blue, and Rest shown in red. (B) Distribution of ICC values between Movie and Rest for each ROI. The red line indicates the conventional threshold from fair to moderate reliability at ICC = 0.4.

The spatial distribution of differences in ICCs did not reveal any marked regional organization (Fig. 4A). For all ROIs, the strength, range, and frequency of Movie and Rest ICC values were highly overlapping (Fig. 4B). Rest distributions had somewhat longer, lower tails than Movie distributions in the DLPFC and TPJ. Across ROIs and conditions, edge ICCs ranged from fair to moderate.

### 3.4 Whole-brain parcel-level multivariate reliability between Movie and Rest

**Fig. 5.**
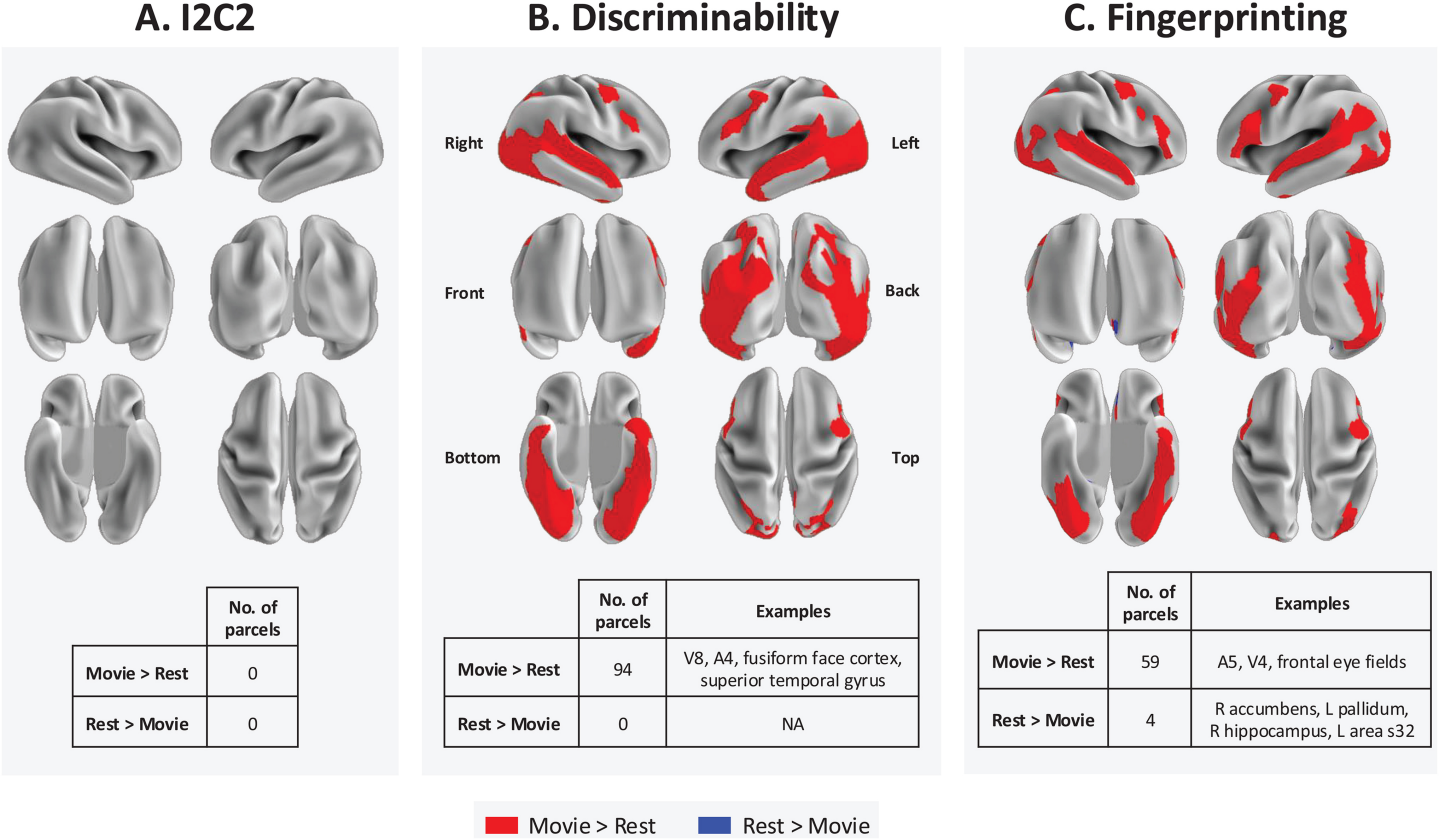
Whole-brain parcel-level multivariate test–retest reliability. Parcels with significantly higher test–retest reliability in Movie than in Rest are shown in red, and those where Rest is higher than Movie are in blue. Statistical significance was determined with permutation testing (5000 permutations per parcel) and FDR corrected across all tests (379 parcels * 3 measures = 1137 tests). Parcels with p < 0.05 after correction are shown. The number of significantly different parcels between movie and rest is summarized below each measure, along with examples of significant regions. No significant differences between conditions were observed with (A) I2C2. Many parcels in visual and temporal regions achieved higher (B) discriminability and (C) fingerprinting accuracy with Movie than with Rest. Four parcels achieved a higher fingerprinting accuracy with Rest, and no parcels were more discriminable with Rest than with Movie. Results can also be visualized interactively athttps://www.headspacestudios.org/resources-reliability.

Repeating the fine-grained multivariate test–retest reliability analysis using each Glasser parcel and subcortical region as an ROI revealed many regional improvements with Movie compared with Rest, and minimal improvements with Rest (Fig. 5). I2C2 did not differ significantly for any parcels between conditions (Fig. 5A). A total of 94 parcels achieved statistically higher discriminability with Movie than with Rest (mean difference in accuracy = 10%, range of differences = 5–18 %), and zero parcels were more discriminable with Rest (Fig. 5B). Many of the parcels with higher discriminability with Movie were located in visual and temporal areas, and none of these parcels were subcortical regions (seeSupplementary Table 1). Similarly, 59 parcels achieved higher fingerprinting accuracy with Movie than with Rest (mean difference in accuracy = 23%, range of differences = 1–47 %), some with differences in accuracy as high as 47% (e.g., bilateral fusiform face complex; seeSupplementary Table 2) (Fig. 5C). Four parcels achieved higher fingerprinting accuracy with Rest than with Movie (mean difference in accuracy = 2.5%, range in differences = 1–7 %; seeSupplementary Table 3), all with small effect sizes (differences in accuracy of 1%) except for the right hippocampus where accuracy was 7% higher for Rest than for Movie. All results are also available as an interactive web-based visualization (R Shiny app) athttps://www.headspacestudios.org/resources-reliability.

## 4 Discussion

We investigated univariate and multivariate reliability of FC collected during movie watching (of concatenated 3–4 minute clips) and resting-state conditions. The goal was to assess whether advantages of movie-fMRI would translate to the ROI level, with a view toward optimizing acquisition conditions for FC-based precision psychiatric research. Overall, the findings from our three psychiatrically relevant ROIs show equivalent performance of Rest and Movie, with some region-specific advantages for Movie in the TPJ. Our findings from the whole-brain analysis reveal many region-specific advantages of movie watching, primarily across visual and temporal regions.

### 4.1 Test–retest reliability in psychiatrically relevant ROIs

Despite its preponderance in the field, we found that resting state does not provide greater reliability of FC relative to movie watching in any measure or in any of the three region’s connectivity profile. Rather, the reliability-based measures collected in these ROIs during movie watching are similar to, or, in some cases, marginally better than rest in this sample.

Discriminability was high across all three regions and conditions despite this ROI-based approach, lending support to the idea that individual differences in a single region’s FC could reliably be used for precision psychiatry. This suggests that although I2C2 (as a stricter measure of reliability) was “only” fair to moderate, the data at the ROI-level are still discriminable. This finding aligns with previous research in rest showing that high identification accuracies are achievable even with a small number of randomly selected edges (Byrge & Kennedy, 2019). Here, we show that this holds true even when using only “isolated” ROIs.

We also found that two of the three multivariate identification-based measures of reliability (discriminability, fingerprinting) generally yielded numerically higher values than the univariate measure (ICC). Although direct comparison of the numerical values of these measures is not feasible across studies due to strong influences of sample characteristics and sample size, the relative findings of higher multivariate reliability than univariate is in line with previous research in rest (Camp et al., 2024;Kragel et al., 2021;Pannunzi et al., 2017). These findings suggest that discrete ROIs, even the relatively small pre-SMA (209 vertices), contain reliable patterns of FC despite low edge-level reliability.

Especially in the context of precision psychiatric research, it is important to note that these encouraging markers of reliability do not necessarily reflect validity or clinical relevance of the FC patterns. For example, noisy data can be highly reliable (Shirer et al., 2015;Zuo & Xing, 2014), but measuring reliable noise is not usually of clinical relevance. It is also possible that the reliability of a region’s FC would be higher or lower than the reliability of a precise target within that region. Though this healthy adult sample with low head motion certainly minimizes some aspects of noise, further research is needed to assess validity at the ROI level, and potentially to determine whether the findings here also relate to the even smaller targets used for some precision psychiatry efforts. Similarly, although identification accuracy reflects the presence of reliable individual differences in FC, it remains unclear whether the individual differences driving successful identification are of clinical importance or utility, and neither high discriminability nor high fingerprinting accuracies are necessary features for future clinical tests or biomarkers. Rather, the hope is that methods that enhance these individual differences will also enhance (or perhaps already include) differences that are subsequently found to be clinically important.

### 4.2 The effect of data amount between Movie and Rest

There appears to be some form of an interaction between condition and data amount. With small amounts of data (i.e., less than 5 minutes), Rest often outperforms Movie reliability, especially with fingerprinting. Ultimately, scans this short are not recommended due to low intersession reliability (Birn et al., 2013;Tomasi et al., 2017). With larger amounts of data, Movie and Rest measures either equalize, or in the TPJ, Movie surpasses Rest. Thus, at recommended data amounts, Movie is either better than, or comparable with, Rest, indicating noninferiority of movie-fMRI. However, in circumstances when longer scans are not feasible, or, in particular regions, Rest may provide higher reliability. The mechanisms underlying the interaction between data amount and condition remain unknown, and future dynamic analyses could help explicate the effects of movie content, frequency distribution of signals, region-specific evoked signals, and other factors relevant to these time-by-reliability curves.

The improvements in reliability with increasing data amount may in part be due to the ability to capture slower changes in FC network configurations, which may be poorly captured with short scans (Birn et al., 2013). The longest scan length studied here was only 11 minutes (680 volumes). For precision psychiatry, more data are generally required. For example, 40 minutes of motion-censored, single-echo data is required for high reliability of the full connectome (Gordon et al., 2017;Gratton et al., 2020;Laumann et al., 2015). Whether Movie would go on to outperform Rest (or vice versa) at higher data amounts remains unknown. While recommendations have been made for data quantity necessary for precision psychiatry with resting-state fMRI (Gratton et al., 2020), additional work would be needed to generate recommendations for movie-fMRI.

### 4.3 Advantages of movie-fMRI in the temporoparietal junction

Our results show that of the three psychiatrically relevant ROIs, the TPJ demonstrates the most improvement of FC measures with movie watching. This finding is interesting given the unique involvement of the TPJ in both low-level and high-level processing relevant to movie watching. At the lower level, the TPJ is involved in the processing of visual and auditory information (Blanke & Arzy, 2005;Igelström & Graziano, 2017;Lopez et al., 2008;Serino et al., 2013). It is possible that the rich audiovisual movie stimulus drives activity in the TPJ in a more reliable and systematic way (relative to resting state). At the higher level, the TPJ has been proposed as a critical hub for our social abilities (Patel et al., 2019), and has also been implicated in language, attention, and theory of mind (Blanke & Arzy, 2005;Igelström & Graziano, 2017;Saxe & Kanwisher, 2003;Wu et al., 2015). Specifically during movie watching, the TPJ is activated by observing others commit errors (Jääskeläinen et al., 2016), watching people engage in nonhabitual actions (Wolf et al., 2018), and perceiving event boundaries (Zacks et al., 2010). Using multiple Hollywood film clips here, rich with social interaction and language, may have further contributed to the improved signal in the TPJ during movie watching in a targeted way. Individual differences in high-level cognitive processes evoked by movies could explain the high interindividual variability in FC observed in the TPJ (Vanderwal et al., 2017) and thus the improved fingerprinting accuracy found here with movies. Overall, the fact that TPJ functions relate so closely to movie stimuli suggests that perhaps the more we drive activity in a given region, the better the reliability and individual differences in that region will be—at least when the region is more proximal to the sensory end of processing streams. Further comparisons using conventional tasks and “region specific” naturalistic paradigms could help.

### 4.4 Reliability of FC from Movie and Rest across the whole brain

Applying this fine-grained multivariate approach to all parcels of the brain revealed many regions where movie watching improved identification-based test–retest reliability measures of FC over rest. This may relate to individual-specific evoked responses to the movies. These regions were mostly located in visual and temporal areas, further supporting the hypothesis that regions whose functions relate more closely to movie watching benefit most from movie-related constraints. This finding is in line with previous research that found improved mean ICC values with movie watching across visual and temporal regions (Wang, Han, et al., 2017). It is important to note that this finding in healthy adults may not generalize to patient populations, but we suggest it might warrant the inclusion of movie-watching runs in fMRI research in patient populations to test this scenario. Perhaps with tailored movie content, movie watching could offer improved reliability in other brain regions. For example, previous research has utilized symptom-related movie content to drive specific brain activity in depression, trichotillomania, psychosis, and Attention-Deficit Hyperactivity Disorder (Gruskin et al., 2020;Lee et al., 2010;Rikandi et al., 2017;Salmi et al., 2020).

To provide some data-based input for decisions surrounding acquisition state for future study design, we have published all whole-brain results as an interactive R Shiny app (https://www.headspacestudios.org/resources-reliability). The app enables exploration of the differences in multivariate test–retest reliability of FC across movie watching and resting state at the parcel level, based on these HCP data.

### 4.5 Practical considerations

We also note other methodological strategies that improve data quality for precision psychiatry and could be combined with the findings presented here. In terms of imaging parameters, multiecho fMRI also improves the test–retest reliability of FC data (Lynch et al., 2020,^2021^). Multiecho data uniquely enable denoising techniques that improve reliability in subcortical regions (many of which are important in psychiatry, such as the SGC) that typically have lower reliability. Thus, combining multiecho with movie-fMRI may offer compounded advantages, improving reliability in both cortical and subcortical regions and improving signal-to-noise ratios via complementary avenues. Within movie-fMRI, tailoring movie choice toward disorder-specific content may also enhance research efforts (Eickhoff et al., 2020;Farb et al., 2011;Gruskin et al., 2020;Rikandi et al., 2017;Salmi et al., 2020). Ongoing work is needed to determine whether these targeted movies improve FC-based data in ways that would impact precision psychiatric research, with comparison both with standard movies like those used here, and with resting state.

### 4.6 Limitations

This study has various limitations that warrant discussion, starting with participant-based limitations. The HCP data used were from healthy participants, but psychiatric populations may differ in factors that affect reliability and fingerprinting such as compliance, arousal, and head motion (Bridgeford et al., 2021;Camp et al., 2024;Horien et al., 2018;Noble et al., 2017;Pardoe et al., 2016;Wang, Han, et al., 2017;Yao et al., 2017). The ROIs were selected specifically for relevance to psychiatric disorders, so findings in those ROIs between healthy and psychiatric populations may be different, which would limit the generalizability. There were also siblings and twins in this sample, which may have lowered the performance of the identification algorithm due to similarities in FC across siblings or twins (Barber et al., 2021;Demeter et al., 2020;Xu et al., 2016;Yang et al., 2016).

Second, some movie-based limitations warrant discussion. Different movie clips were used in the two movie scans. Thus, our findings likely underestimate the potential reliability of movie-fMRI under actual test–retest conditions. Further, the movie clips were all quite short (~3.5–4.5 minutes), and using longer narratives has been shown to engage the brain differently (Hasson et al., 2008,^2015^;Jääskeläinen et al., 2021), so results may not be consistent across longer movies, especially in the default network and higher order cortical areas. There may also be an interaction between movie content and psychiatric diagnosis that is not possible to investigate in this sample of healthy adults.

Third, we chose to use connectome-based fingerprinting to estimate the reliability of individual differences in FC. This method is influenced by sample size, scan length, and age (Horien et al., 2018), and would ideally be complemented by another measure such as predictive modeling and additional efforts to assess validity, most of which were not feasible with this sample size.

Fourth, we used minimally preprocessed data and did not apply rigorous denoising techniques or test reliability measures across denoising manipulations. Although preprocessing has major impacts on the reliability of FC (Noble et al., 2019;Shirer et al., 2015), we chose to limit preprocessing for this healthy, young sample due to the low observed motion and the cross-condition analysis (where data from both conditions are being preprocessed in the same way). Nonetheless, differences in physiological factors (e.g., respiration) may differ systematically between movies and rest and are potential confounds that warrant future investigation. These results should be taken as a general benchmark that can be built upon with future research tailored to specific populations and preprocessing questions.

Lastly, this dataset was collected with a 7T fMRI scanner. While 7T scanners are becoming more common, the vast majority of research, especially clinical scans, are most likely being conducted on 3-Tesla scanners. Differences in resolution and signal-to-noise in specific regions may thus affect our results in specific ways (Mandelkow et al., 2017).

### 4.7 Main conclusions

1. In most brain regions and across all reliability-based measures, movie and rest were comparable despite the use of different movies across test–retest scans.
2. With movie-watching data, 25% of regions had better discriminability and 15% had better fingerprinting relative to rest. In the TPJ (an a priori ROI), movie watching improved both univariate and multivariate measures of reliability relative to rest.
3. With resting-state data, 1% of parcels had better fingerprinting than movie. In our three psychiatric ROIs, reliability measures were better for rest at subrecommended data amounts (i.e., less than 5 minutes).
4. These findings suggest that reliability is state dependent in some regions, and that movie watching may provide a useful alternative to rest for the collection of reliable and discriminable measures of FC.

## Data and Code Availability

Original data used in this study are available at the HCP website (https://db.humanconnectome.org/). Code used to perform these analyses is available at our Github repository:https://github.com/tvanderwal/roi_reliability_shearer. Whole-brain results are available athttps://www.headspacestudios.org/resources-reliability.

## Author Contributions

H.S.—conceptualization, data curation, formal analysis, funding acquisition, methodology, visualization, writing—original draft, writing—review & editing. J.E.—data curation, formal analysis, methodology, software. F.V.R.—supervision, writing—review & editing. S.N.—writing—review & editing. T.X.—methodology. T.V. —conceptualization, methodology, supervision, writing—review & editing, funding acquisition.

## Declaration of Competing Interest

F.V.R. has received in-kind equipment support for an investigator-initiated trial from MagVenture. He has received honoraria for participation in an advisory board for Allergan. F.V.R. is a volunteer director on the board of directors for the British Columbia Schizophrenia Society. The other authors declare that they have no known competing financial interest or personal relationships that could have appeared to influence the work reported in this paper.

## Supplementary Material

Supplementary Material

## Citation Bias reporting

Recent work in several fields of science has identified a bias in citation practices such that papers from women and other minority scholars are undercited relative to the number of such papers in the field. Here we sought to proactively report the predicted gender and race of the first and last author of each reference using available tools and databases (www.gender-api.com,https://github.com/appeler/ethnicolr,https://github.com/dalejn/cleanBib). Excluding self-citations to the first and last authors of our current paper, our references contain 10.96% woman(first)/woman(last), 12.48% man/woman, 24.55% woman/man, and 52.01% man/man. The gender prediction is limited in that (a) names, pronouns, and social media profiles used to construct the databases may not, in every case, be indicative of gender identity and (b) it cannot account for intersex, nonbinary, or transgender people. Our references also contain 13.23% author of color (first)/author of color(last), 16.28% white author/author of color, 22.46% author of color/white author, and 48.03% white author/white author. The race prediction is limited in that (a) names and Florida Voter Data to make the predictions may not be indicative of racial/ethnic identity, and (b) it cannot account for Indigenous and mixed-race authors, or those who may face differential biases due to the ambiguous racialization or ethnicization of their names.
