# Supplementary material for: Comparing reliability-based measures of functional connectivity between movie and rest: An ROI-based approach
Source: Imaging Neurosci (Camb). 2025 Jan 2;3:imag_a_00411. doi: 10.1162/imag_a_00411 (PMC12319993; doi:10.1162/imag_a_00411)
Supplement: Supplementary Material [file imag_a_00411-supp.pdf]

Supplementary Materials

Parcel #	Parcel label	Corrected p	Movie	Rest	Movie - Rest
2	L_MST	0.01	0.902	0.813	0.088
5	L_V3	0.009	0.894	0.806	0.088
6	L_V4	0	0.935	0.821	0.114
7	L_V8	0	0.91	0.732	0.178
11	L_PEF	0.007	0.973	0.908	0.065
12	L_55b	0.013	0.965	0.908	0.057
13	L_V3A	0.016	0.889	0.795	0.094
16	L_V7	0.045	0.899	0.823	0.076
18	L_FFC	0	0.949	0.805	0.144
19	L_V3B	0	0.926	0.812	0.113
20	L_LO1	0	0.935	0.82	0.116
21	L_LO2	0	0.942	0.823	0.119
22	L_PIT	0	0.93	0.777	0.153
23	L_MT	0	0.877	0.769	0.108
25	L_PSL	0	0.969	0.888	0.081
28	L_STV	0	0.969	0.855	0.114
48	L_LIPv	0	0.943	0.878	0.065
50	L_MIP	0.041	0.953	0.892	0.06
79	L_IFJa	0.01	0.963	0.892	0.072
80	L_IFJp	0.028	0.956	0.897	0.059
81	L_IFSp	0.005	0.96	0.889	0.071
95	L_LIPd	0.003	0.954	0.89	0.064
105	L_PFcm	0.027	0.872	0.796	0.075
107	L_TA2	0	0.805	0.699	0.106
123	L_STGa	0	0.857	0.737	0.121
125	L_A5	0	0.918	0.782	0.136
127	L_PHA3	0.01	0.887	0.817	0.07
128	L_STSda	0	0.943	0.799	0.144
129	L_STSdp	0	0.936	0.834	0.103
130	L_STSvp	0.018	0.937	0.875	0.063
131	L_TGd	0.005	0.836	0.762	0.074
135	L_TF	0	0.835	0.704	0.131
136	L_TE2p	0	0.902	0.813	0.089
137	L_PHT	0.009	0.974	0.919	0.055
138	L_PH	0	0.945	0.831	0.113

139	L_TPOJ1	0	0.951	0.842	0.109
140	L_TPOJ2	0	0.966	0.882	0.083
141	L_TPOJ3	0.01	0.948	0.869	0.079
142	L_DVT	0.01	0.946	0.871	0.076
143	L_PGp	0.029	0.966	0.907	0.059
146	L_IP0	0.012	0.96	0.888	0.072
154	L_VMV3	0	0.9	0.741	0.159
156	L_V4t	0	0.934	0.782	0.152
157	L_FST	0.003	0.921	0.83	0.091
158	L_V3CD	0.003	0.933	0.841	0.093
159	L_LO3	0	0.943	0.813	0.13
160	L_VMV2	0.003	0.867	0.765	0.102
163	L_VVC	0	0.913	0.782	0.13
172	L_TGv	0	0.82	0.726	0.094
175	L_A4	0	0.889	0.735	0.154
176	L_STSva	0.01	0.921	0.847	0.074
182	R_MST	0	0.889	0.79	0.099
185	R_V3	0.032	0.893	0.816	0.077
186	R_V4	0	0.935	0.819	0.115
187	R_V8	0	0.893	0.723	0.169
190	R_FEF	0.005	0.956	0.882	0.074
192	R_55b	0.021	0.971	0.917	0.054
196	R_V7	0.049	0.913	0.838	0.075
198	R_FFC	0	0.943	0.8	0.143
199	R_V3B	0.01	0.926	0.834	0.092
200	R_LO1	0	0.932	0.812	0.12
201	R_LO2	0	0.922	0.796	0.127
202	R_PIT	0	0.916	0.795	0.121
203	R_MT	0	0.894	0.773	0.121
207	R_PCV	0.016	0.934	0.854	0.079
208	R_STV	0	0.911	0.821	0.09
230	R_MIP	0.007	0.953	0.882	0.071
259	R_IFJa	0.009	0.965	0.893	0.073
260	R_IFJp	0	0.951	0.864	0.087
275	R_LIPd	0.005	0.954	0.888	0.066
287	R_TA2	0.021	0.727	0.654	0.073
303	R_STGa	0	0.839	0.713	0.126
305	R_A5	0	0.899	0.772	0.127
307	R_PHA3	0.016	0.882	0.807	0.076

308	R_STSda	0	0.913	0.806	0.107
309	R_STSdp	0	0.953	0.856	0.097
315	R_TF	0	0.831	0.713	0.118
316	R_TE2p	0.007	0.906	0.835	0.071
318	R_PH	0	0.927	0.816	0.111
319	R_TPOJ1	0	0.951	0.854	0.098
320	R_TPOJ2	0.003	0.945	0.868	0.078
321	R_TPOJ3	0.012	0.94	0.864	0.075
322	R_DVT	0.047	0.939	0.876	0.063
326	R_IP0	0.037	0.957	0.894	0.063
333	R_VMV1	0.033	0.86	0.781	0.079
334	R_VMV3	0	0.881	0.738	0.143
336	R_V4t	0	0.908	0.784	0.125
337	R_FST	0	0.922	0.811	0.111
338	R_V3CD	0	0.946	0.853	0.093
339	R_LO3	0	0.944	0.818	0.125
340	R_VMV2	0.013	0.866	0.783	0.083
343	R_VVC	0	0.926	0.79	0.136
352	R_TGv	0.035	0.747	0.68	0.067
355	R_A4	0	0.883	0.732	0.151

Table 1. Discriminability: parcels with significantly higher discriminability with Movie than Rest. Parcel # and label are from the Glasser parcellation. Corrected p values are FDR corrected across all 1,137 tests (360 parcels + 19 subcortical regions, 3 multivariate measures).

Parcel #	Parcel label	Corrected p	Movie	Rest	Movie - Rest
6	L_V4	0	0.642	0.339	0.303
7	L_V8	0	0.33	0.11	0.22
11	L_PEF	0.048	0.615	0.431	0.183
12	L_55b	0.028	0.688	0.505	0.183
18	L_FFC	0	0.642	0.174	0.468
19	L_V3B	0	0.651	0.376	0.275
20	L_LO1	0	0.578	0.303	0.275
21	L_LO2	0	0.541	0.294	0.248
22	L_PIT	0	0.459	0.165	0.294
25	L_PSL	0	0.734	0.468	0.266
28	L_STV	0	0.688	0.44	0.248
74	L_44	0.022	0.725	0.55	0.174

75	L_45	0.047	0.761	0.596	0.165
79	L_IFJa	0.013	0.679	0.477	0.202
81	L_IFSp	0	0.706	0.486	0.22
107	L_TA2	0.035	0.138	0.018	0.119
123	L_STGa	0	0.275	0.055	0.22
125	L_A5	0	0.413	0.119	0.294
128	L_STSda	0	0.596	0.211	0.385
129	L_STSdp	0.003	0.505	0.257	0.248
135	L_TF	0.04	0.165	0.046	0.119
138	L_PH	0	0.578	0.33	0.248
139	L_TPOJ1	0.003	0.578	0.349	0.229
150	L_PGi	0.046	0.771	0.606	0.165
154	L_VMV3	0.015	0.312	0.128	0.183
157	L_FST	0.028	0.459	0.284	0.174
158	L_V3CD	0.009	0.569	0.339	0.229
159	L_LO3	0.005	0.541	0.284	0.257
163	L_VVC	0	0.468	0.147	0.321
164	L_25	0	0.018	0.009	0.009
172	L_TGv	0.019	0.211	0.064	0.147
175	L_A4	0.007	0.257	0.073	0.183
186	R_V4	0	0.624	0.257	0.367
187	R_V8	0.003	0.367	0.156	0.211
190	R_FEF	0.016	0.661	0.45	0.211
192	R_55b	0	0.761	0.55	0.211
198	R_FFC	0	0.633	0.174	0.459
199	R_V3B	0.005	0.532	0.321	0.211
200	R_LO1	0.04	0.468	0.284	0.183
201	R_LO2	0.013	0.367	0.147	0.22
202	R_PIT	0	0.394	0.11	0.284
203	R_MT	0.047	0.312	0.147	0.165
255	R_45	0.024	0.798	0.633	0.165
260	R_IFJp	0.01	0.541	0.321	0.22
261	R_IFSp	0.032	0.734	0.578	0.156
275	R_LIPd	0.003	0.569	0.358	0.211
305	R_A5	0	0.303	0.073	0.229
308	R_STSda	0	0.367	0.11	0.257
309	R_STSdp	0.005	0.56	0.33	0.229
318	R_PH	0.019	0.422	0.239	0.183
319	R_TPOJ1	0.012	0.505	0.303	0.202

321	R_TPOJ3	0.024	0.486	0.312	0.174
325	R_IP1	0.007	0.78	0.587	0.193
326	R_IP0	0.018	0.688	0.495	0.193
334	R_VMV3	0	0.321	0.083	0.239
336	R_V4t	0	0.422	0.156	0.266
338	R_V3CD	0.009	0.578	0.376	0.202
343	R_VVC	0	0.486	0.202	0.284
355	R_A4	0.016	0.257	0.083	0.174

Table 2. Fingerprinting: Parcels with significantly higher fingerprinting with Movie than Rest. Parcel # and label are from the Glasser parcellation. Corrected p values are FDR corrected across all 1,137 tests (360 parcels + 19 subcortical regions, 3 multivariate measures).

Parcel #	Parcel label	Corrected p	Movie	Rest	Movie - Rest
165	L_s32	0.003	0.00917431	0.018	-0.009
300	R_H	0.044	0.00917431	0.083	-0.073
362	accumbens_right	0	0	0.009	-0.009
373	pallidum_left	0	0	0.009	-0.009

Table 3. Fingerprinting: parcels with significantly higher fingerprinting with Rest than Movie. Parcel # and label are from the Glasser parcellation. Corrected p values are FDR corrected across all 1,137 tests (360 parcels + 19 subcortical regions, 3 multivariate measures).
